# Ovarian intelligence: AI applications leveraging AMH and inhibin B

**DOI:** 10.3389/fendo.2026.1724929

**Published:** 2026-05-11

**Authors:** Huiyu Xu, Farideh Bischoff, Qiang Wang, Rong Li

**Affiliations:** 1 State Key Laboratory of Female Fertility Promotion, Center for Reproductive Medicine, Department of Obstetrics and Gynecology, Peking University Third Hospital, Beijing, China; 2 National Clinical Research Center for Obstetrics and Gynecology (Peking University Third Hospital), Beijing, China; 3 Key Laboratory of Assisted Reproduction (Peking University), Ministry of Education, Beijing, China; 4 Beijing Key Laboratory of Reproductive Endocrinology and Assisted Reproductive Technology, Beijing, China; 5 National Clinical Key Specialty Construction Program, Beijing, China; 6 HerAnova Lifesciences, Inc., Boston, MA, United States; 7 Institute of Infection, Immunology and Tumor Microenvironment, Hubei Province Key Laboratory of Occupational Hazard Identification and Control, Medical College, Wuhan University of Science and Technology, Wuhan, China

**Keywords:** AI, AMH, inhibin B, OvaRePred, ovarian intelligence, PCOSt, POvaStim

## Abstract

Artificial intelligence is transforming reproductive medicine by enabling more nuanced and personalized interpretation of key biomarkers such as anti-Müllerian hormone (AMH) and inhibin B. While AMH is widely adopted for assessing ovarian reserve, inhibin B—an follicle-stimulating hormone (FSH)-dependent marker of follicular activity—has been historically underutilized due to its dynamic nature and lack of standardized assays. In this review, we explore how AI-driven tools can integrate these hormonal signals to improve clinical decision-making. We highlight three representative models: OvaRePred (also known as HerTempo), which predicts ovarian reserve and perimenopausal timing; PCOSt, which enables early screening and phenotyping of polycystic ovary syndrome; and POvaStim, which personalizes gonadotropin dosing by modeling ovarian sensitivity. Collectively, these tools offer new possibilities for individualized fertility management and may help shift reproductive care from reactive treatment toward proactive, data-informed health planning. We also discuss emerging innovations such as cross-platform assay harmonization, point-of-care hormone testing, and longitudinal biomarker modeling. Looking ahead, integration with nutritional interventions, wearable technologies, and genetic or immunologic data could extend these tools beyond assisted reproduction—supporting a broader, AI-enabled ecosystem for women’s lifelong health.

## 1 Introduction: from static markers to adaptive forecasts

Ovarian reserve—the finite pool of oocytes historically gauged by follicle-stimulating hormone (FSH), estradiol and antral−follicle count (1–6)—remains the cornerstone of female fertility assessment. Anti−Müllerian hormone (AMH), with its minimal cycle variability (7–10), revolutionized reserve evaluation, while inhibin B provides dynamic, FSH−driven feedback on follicular activity (11, 12). Yet no single biomarker fully addresses patients’ pressing concerns: How rapidly is my reserve depleting? When will perimenopause begin? Can a simple blood draw determine whether I have Polycystic Ovary Syndrome (PCOS)? What are the optimal starting and adjustment doses for my ovarian−stimulation protocol? AI now bridges these gaps by integrating hormone assays, imaging metrics and patient demographics into continuously learning prediction engines.

In this review, we (i) explore how AMH and inhibin B function as “stock” and “flow” inputs for reproductive AI platforms, (ii) showcase the real−world impact of flagship tools such as OvaRePred (also known as HerTempo), PCOSt and POvaStim, and (iii) discuss future directions for refining these algorithms and embedding predictive forecasts into routine clinical care.

## 2 Essentials of AMH and inhibin B for AI engineers

Accurate, data-driven models of ovarian function hinge on two complementary biomarkers: AMH and inhibin B. Below are the key properties of each signal and guidance on how to integrate them into predictive algorithms.

### 2.1 AMH: a stable “stock” signal of follicle quantity

AMH is secreted by granulosa cells in small growing follicles—from the primary stage through early antral stages—largely independent of gonadotropin (Gn) fluctuations (7, 8, 10, 13). Primordial follicles—the ovary’s most immature stage—do not secrete AMH (14, 15). Secretion begins once they become primary follicles and continues in a gonadotropins (Gn)-independent fashion through the secondary and pre-antral stages, as granulosa cells remain unresponsive to FSH and LH (10). This constant output underlies AMH’s minimal variability across the menstrual cycle, making it a robust ovarian-reserve marker. At the small antral stage (2–8 mm), follicles gain gonadotropin sensitivity, and AMH production becomes FSH-modulated, which explains the modest cycle-related fluctuations observed (16–18).

Which follicles drive circulating AMH remains debated. Weenen et al. reported weak AMH in most primary follicles, peaking in secondary, pre-antral, and small antral follicles ≤4 mm before tapering in larger (4–8 mm) follicles (10). Conversely, Jeppesen et al. found that 5–8 mm follicles contribute most to serum AMH (19). These contrasting findings may account for why some women experience larger AMH swings than others and suggest that both Gn-independent and Gn-dependent small follicles play roles in hormone dynamics.

#### 2.1.1 Strengths

##### 2.1.1.1 Cycle stability

Intra-individual variation in AMH levels within and across menstrual cycles is minimal—typically less than 10% (20). This stability arises because AMH is predominantly secreted by granulosa cells in small pre-antral and early antral follicles, largely independent of gonadotropin fluctuations (10). Although minor, Gn-dependent modulation during late follicular phases can produce slight AMH variations, these changes are rarely clinically significant. Significant AMH fluctuations occur mainly under conditions of substantial estradiol elevation, such as ovarian stimulation (21) or ovarian cysts (22), though these cases are uncommon. Consequently, a single AMH measurement reliably captures baseline ovarian reserve, streamlining fertility assessment (23).

##### 2.1.1.2 Sampling accuracy and flexibility

Over the past decade, AMH measurement has evolved from variable manual enzyme−linked immunosorbent assays (ELISAs) to fully automated chemiluminescent immunoassays, achieving inter− and intra−assay coefficients of variation below 5% and markedly improving calibration consistency and assay reproducibility. Concurrently, the advent of microfluidic platforms and dried blood spot (DBS) methodologies has enabled serum−equivalent quantification of AMH from minimal finger−prick and menstrual fluid samples, (24, 25) supporting low−volume point−of−care (POC) and at−home workflows for ovarian−reserve assessment and longitudinal monitoring. Moreover, AMH’s demonstrated stability across diverse storage and transport conditions underpins26 decentralized reproductive−health programs and expedited fertility counseling—eliminating routine venipuncture and centralized laboratories while enhancing patient comfort, accessibility, and individualized care.

##### 2.1.1.3 Predictive correlation

Serum AMH concentrations reliably reflect ovarian reserve and strongly correlate with reproductive outcomes, particularly oocyte yield in IVF treatments and timing of menopause onset (5, 26–28). Incorporating AMH into predictive algorithms allows clinicians to accurately counsel patients regarding fertility potential, tailor stimulation protocols, and anticipate menopause, thus enhancing personalized reproductive management.

#### 2.1.2 Limitations

Limitations of AMH as a reproductive biomarker require careful consideration in both clinical and research contexts due to biological and technical challenges.

##### 2.1.2.1 Pathological sensitivity

Certain ovarian conditions can significantly distort AMH measurements. For example, granulosa-cell tumors produce unusually high AMH levels (29), falsely suggesting adequate ovarian reserve and potentially delaying accurate diagnosis. PCOS also elevates AMH levels due to increased follicle numbers (30), though this does not reflect true reproductive potential, complicating the interpretation of ovarian reserve. Thus, AMH results in these conditions should always be supplemented with additional diagnostic tools like ultrasonography and clinical assessments (31).

##### 2.1.2.2 Contraceptive effects

Combined oral contraceptives (COCs) transiently suppress follicle development, reducing serum AMH concentrations and falsely indicating lower ovarian reserve (32). Levels typically normalize two months after cessation, prompting guidelines that recommend delaying AMH testing following contraceptive use (32, 33). If immediate discontinuation of contraception is impractical, clinicians must interpret AMH results conservatively and consider re-testing.

##### 2.1.2.3 Assay drift and standardization issues

Technical variability among AMH assays poses substantial interpretation challenges (34). Differences across commercial immunoassays, antibody specificity, calibration methods, and even batch-to-batch inconsistencies within the same assay complicate clinical decision-making and data comparability in research (34, 35). Accurate interpretation therefore demands rigorous quality control, standardization protocols, and transparent documentation of assay types. Clinical interpretation must integrate AMH data thoughtfully within broader diagnostic frameworks, including patient age, antral follicle count, and clinical history, to mitigate these limitations effectively (33).

### 2.2 Inhibin B: an FSH-dependent “flow” signal of follicular activity

**Figure 1:**
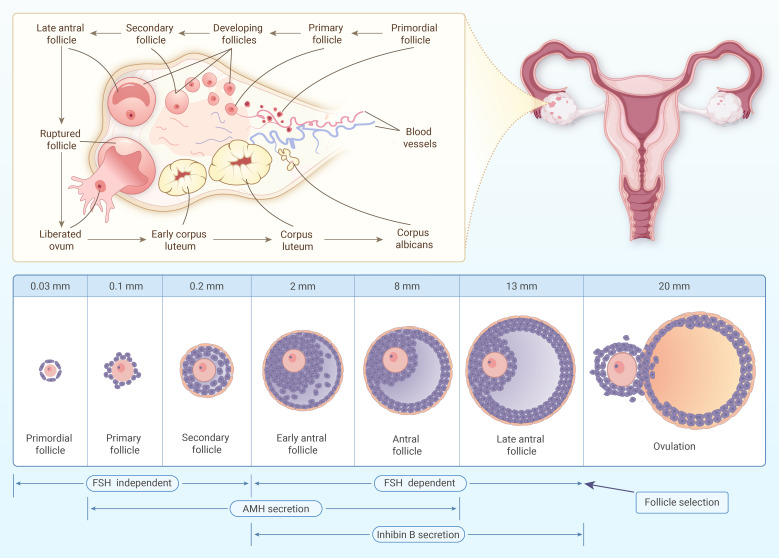
Schematic diagram illustrating the secretion of AMH and inhibin B. The stages of follicle development in the ovary, from the primordial follicle to ovulation, are shown. AMH is secreted from primary to early antral follicles, involving both FSH-dependent and FSH-independent phases, and plays a crucial role in assessing ovarian reserve. Inhibin B secretion begins at the early antral follicle stage and is FSH-dependent, providing feedback regulation of FSH levels. The diagram also highlights the transition to follicle selection, culminating in the ovulation of the dominant follicle.

Inhibin B is produced by FSH-stimulated granulosa cells within mid-sized antral follicles, typically ranging from approximately 3 to 13 mm in diameter (36). Its secretion profile is summarized in **Figure 1**, alongside AMH. Inhibin B levels reach their peak around cycle day 7, just prior to the selection of the dominant follicle (12, 36). Following this peak, levels decline rapidly as estradiol production rises, signaling the transition toward follicular dominance and the ovulatory phase.

**Figure 2:**
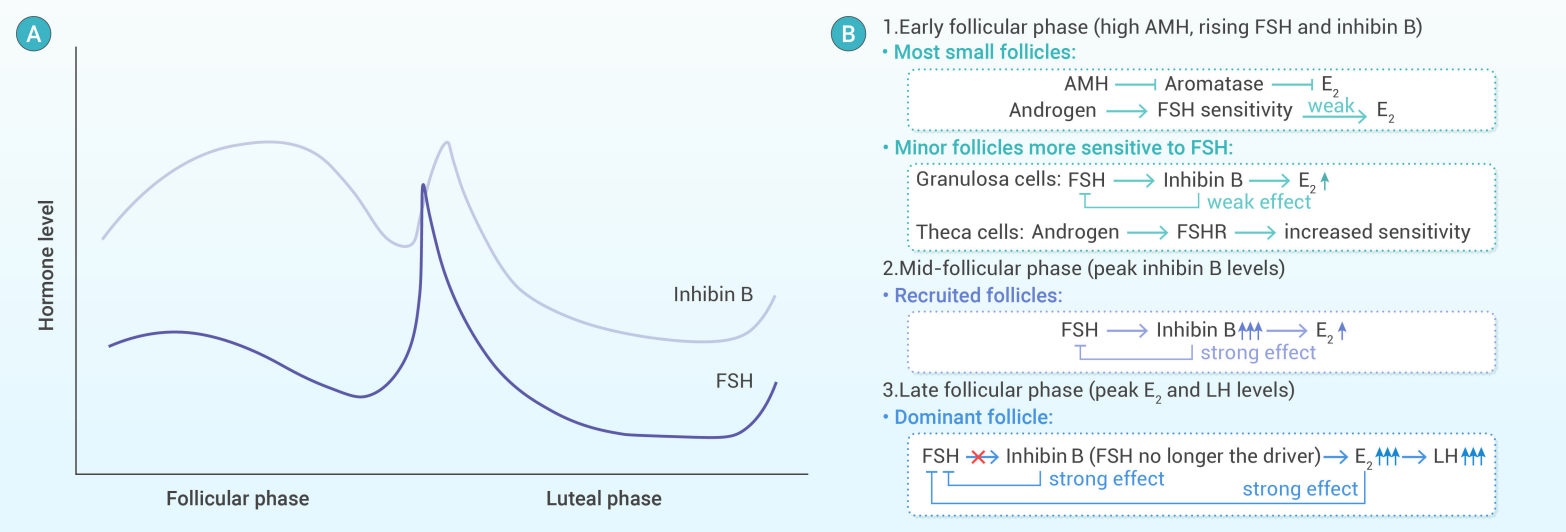
Hormonal regulation during the follicular phase. **(A)** Hormonal fluctuations during the menstrual cycle: The graph illustrates the dynamic changes in FSH and inhibin B levels across the follicular and luteal phases. Inhibin B peaks in the early-to-mid follicular phase, corresponding to FSH to support follicular recruitment. This figure is a redesigned adaptation based on the original illustration from “Inhibin B - An Overview” (ScienceDirect Topics), available at https://www.sciencedirect.com/topics/nursing-and-health-professions/inhibin-b. **(B)** Regulation of ovarian function: The diagram depicts the complex hormonal interactions during different phases of the follicular phase. AMH and androgens regulate the follicular response to FSH, while inhibin B and estradiol (E_2_) provide feedback to modulate FSH secretion, ensuring proper follicular development and ovulation.

Given its direct dependence on circulating FSH concentrations, inhibin B serves as a sensitive and dynamic biomarker for assessing the immediate responsiveness of ovarian follicles to hormonal stimulation (12). Its temporal profile closely mirrors FSH-driven follicular recruitment, making it particularly useful for real-time monitoring during controlled ovarian stimulation protocols. The relationship between inhibin B and FSH is illustrated in **Figure 2**.

#### 2.2.1 Strengths: dynamic response and predictive value

Serial measurement of inhibin B—such as evaluating changes between early follicular phase time points (e.g., cycle day 2 and day 6)—provides clinically valuable insight into the ovary’s immediate responsiveness to both endogenous and exogenous FSH stimulation (37–39). In contrast to static biomarkers, this dynamic assessment allows clinicians to determine early in the stimulation process whether follicles are responding appropriately, thereby enabling timely protocol refinement to improve clinical outcomes. In this context, dynamic response does not denote a closed physiological feedback loop; rather, it refers to the clinical interpretation of sequential inhibin B changes as near-real-time indicators of follicular activity.

The extent of inhibin B change across early stimulation has shown strong predictive value for assisted reproductive technology (ART) outcomes. Specifically, this delta index is closely associated with the number of oocytes ultimately retrieved and helps identify patients at increased risk of poor ovarian response (POR) (37, 38, 40). Thus, inhibin B dynamics provide a practical and quantifiable tool for individualizing ovarian stimulation strategies.

Within controlled ovarian stimulation (COS), ongoing monitoring of inhibin B therefore represents a useful adjunct to traditional clinical and ultrasound evaluation (37, 38, 40–42). Early increases in inhibin B reflect FSH-driven activation of small antral follicles and are consistently correlated with subsequent ovarian response and oocyte yield (37, 38). Moreover, serial inhibin B trends often precede or complement ultrasound findings, adding an additional biological dimension to decision-making. When interpreted alongside other clinical indicators, inhibin B trajectories can help clinicians recognize insufficient or excessive stimulation earlier, supporting safer and more effective intra-cycle dose adjustments.

#### 2.2.2 Limitations

##### 2.2.2.1 Sampling intensity

Due to inhibin B’s rapid kinetics and highly dynamic nature during the early follicular phase, accurate interpretation requires at least two carefully timed blood draws per menstrual cycle (e.g., cycle days 2 and 6). This necessity significantly increases patient inconvenience, logistical complexity, and healthcare resource demands, potentially restricting its routine clinical adoption. Additionally, variability in timing can impact result consistency, necessitating meticulous scheduling and patient compliance.

##### 2.3.2.2 Under-utilization

Historically, inhibin B was frequently assessed at single, isolated time points, disregarding its inherently dynamic fluctuations (43, 44). Such limited sampling approaches failed to capture critical changes reflective of follicular responsiveness and consequently undervalued inhibin B’s predictive potential. As a result, numerous legacy datasets inadequately represent the biomarker’s full clinical utility, thereby impeding widespread recognition and integration into standard fertility assessment protocols.

##### 2.2.2.3 Assay standardization

Similar to AMH, inhibin B assay results exhibit variability across different analytical platforms, complicating direct comparisons between laboratories and clinical studies (45). However, fewer manufacturers currently produce validated, high-throughput inhibin B kits, further constraining assay standardization and widespread implementation. This variability necessitates rigorous calibration efforts and complicates multi-center research, ultimately limiting the biomarker’s broader clinical integration and acceptance in reproductive medicine.

### 2.3 Complementarity: combining “stock” and “flow” for richer AI features

AMH and inhibin B offer distinct yet complementary insights into ovarian function, significantly enhancing predictive analytics when integrated within artificial intelligence (AI) models. AMH primarily represents ovarian “stock,” indicating the number of remaining follicles, and is relatively stable both across and within menstrual cycles (10, 19, 20). Its stable concentration reflects a static biological resource, largely independent of immediate hormonal fluctuations. Conversely, inhibin B functions as a “flow” indicator, rapidly changing in response to FSH levels and follicular dynamics, thus reflecting real-time ovarian activity (12).

The complementary nature of these biomarkers is underscored by their differing temporal behaviors and biological drivers. As shown in **Table 1**, AMH levels remains consistent enough that a single measurement reliably assesses baseline ovarian reserve, while inhibin B’s rapid surges and declines necessitate serial measurements to accurately capture the ovary’s dynamic responsiveness. Hence, AMH addresses the primary question of “how much ovarian reserve remains?”, whereas inhibin B answers, “how actively is the ovary currently responding?”.

**Table 1:** Complementarity: combining “stock” and “flow” for richer AI features.

Property	AMH (“stock”)	Inhibin B (“flow”)
Temporal behaviour	Relatively stable across and within cycles	Surges and falls within days/hours
Biological driver	Follicle quantity	FSH-driven follicle activity
Primary question	“How much reserve remains?”	“How strongly is the ovary working?”
Sampling advantage	Single sample; minimal timing bias	Requires early-cycle or stimulus-cycle serial sampling

## 3 Flagship AI applications

Here we highlight several flagship applications built primarily around AMH and inhibin B, illustrating how these biomarkers can be transformed into actionable intelligence to support ovarian reserve assessment, PCOS risk stratification, and individualized dosing strategies in ART. Importantly, the algorithmic cores of these platforms rely on transparent, well-established statistical models such as logistic and linear regression, rather than opaque black-box architectures or computation-intensive deep learning systems. This design choice significantly reduces computational burden, facilitates implementation across different clinical environments, and enhances interpretability and regulatory acceptability. Despite their computational simplicity, these tools leverage large-scale real-world datasets to produce individualized probability outputs instead of fixed diagnostic cut-offs, enable iterative performance optimization as new data accumulate, and can be embedded within intelligent digital ecosystems that support scalable, personalized decision-making. In this sense, these tools are best viewed as AI-enabled clinical decision-support platforms that occupy a pragmatic middle ground between traditional biostatistics and fully autonomous AI, combining methodological robustness, efficiency, and broad clinical deployability.

### 3.1 OvaRePred (HerTempo): ovarian reserve scoring and peri-menopause-age forecasting

Ovarian reserve remains the single most critical determinant of a woman’s reproductive potential, yet its evaluation remains inconsistent and fragmented in clinical practice. Current assessments typically rely on isolated markers such as AMH, AFC, or FSH. However, these indicators are often interpreted qualitatively or with arbitrary thresholds (33, 46), lacking standardized scoring systems or longitudinal context. This leads to inconsistent interpretation, missed early decline, and poorly timed fertility decisions. A unified, quantitative, and future-oriented tool is urgently needed to enable precise evaluation and reproductive planning.

OvaRePred (HerTempo) (demo: http://121.43.113.123:8005/) is a flagship AI tool designed to provide individualized ovarian reserve assessment and menopause-age prediction through a logistic-regression framework. It includes three scalable model variants tailored to different data-availability scenarios: the AA model (AMH + age), the AFA model (AMH + FSH + age), and the AAFA model (AMH + FSH + AFC + age). Importantly, results from our large real-world ART datasets demonstrate that AMH is the dominant predictive contributor, accounting for ≈95% of the AA model, while retaining superior practicality because blood sampling for AMH can be performed on any day of the menstrual cycle, greatly expanding accessibility and enabling application in routine health management and non-ART settings (3, 28, 47–50). The AA model then feed into a sigmoid-shaped ovarian aging curve derived from population-level ART data, enabling nuanced interpretation of ovarian functional decline. It produces three key outputs: (i) a 1–100 ovarian reserve score, (ii) an individualized ovarian endocrine age that maps a woman’s current ovarian reserve to a normative aging curve, and (iii) predicted ages at which diminished ovarian reserve (score = 50) and perimenopause onset (score minimum) are expected to occur. This allows for both present-state profiling and future-state forecasting.

Because OvaRePred (HerTempo) predicts the age at which specific ovarian functional states are reached, clear operational definitions of these milestones are essential. Diminished ovarian reserve (DOR) does not have a universally accepted clinical definition and is variably described using combinations of AMH, AFC, basal FSH thresholds, or ovarian response criteria in ART. In our modeling framework, DOR is defined quantitatively as the ovarian reserve score of 50, corresponding to a 50% predicted probability of poor ovarian response. This probability-based threshold anchors the concept of DOR to a clinically meaningful outcome rather than to a single biomarker cutoff. Importantly, because the model forecasts the age at which a given ovarian reserve probability is expected to occur, each milestone must correspond to a defined risk level. Thus, the DOR age in this context represents the predicted age at which the likelihood of poor ovarian response reaches 50%, providing a continuous and outcome-oriented interpretation of ovarian functional decline.

Similarly, the predicted “perimenopause onset age” generated by OvaRePred should be interpreted within the constraints of the underlying dataset. As the model was developed using large-scale ART populations, truly menopausal women are not represented in the training cohort. Therefore, classical menstrual staging systems, such as STRAW-based definitions of perimenopause, cannot be directly applied. Instead, in this framework, the predicted perimenopause milestone corresponds to the age at which the ovarian reserve score reaches the lowest range observed within the ART population, reflecting women with extremely poor ovarian reserve who are biologically proximate to the menopausal transition. This represents a pragmatic and function-based proxy rather than a formal diagnostic redefinition of perimenopause. We emphasize that these milestones are model-derived operational definitions designed to enable longitudinal forecasting of ovarian functional aging. They complement, rather than replace, established clinical criteria and provide a probability-anchored structure for individualized reproductive counseling and anticipatory health planning.

It is important to clarify that, in the context of ART populations, this “peri-menopause onset” does not denote true menopause, but rather corresponds to the subgroup with the poorest ovarian reserve who are biologically near the menopausal transition. This represents a pragmatic and clinically meaningful surrogate necessitated by the fact that truly menopausal women are not included in ART datasets.

Beyond static evaluation, OvaRePred incorporates population-level ovarian-aging dynamics. Based on established ovarian reserve models, we characterized the increasing probability of diminished ovarian reserve (DOR) across age and identified a relatively stable pattern of ovarian depletion at the population level. Consistent with the “Fixed Interval” hypothesis, although individual variability exists, an individual woman’s ovarian aging trajectory can be meaningfully inferred from population-derived decline patterns. Thus, knowing both the current ovarian state and the population-derived aging velocity allows interval-based forecasting of when ovarian reserve will reach specific clinically relevant thresholds.

OvaRePred (HerTempo) has demonstrated excellent calibration performance, with an aging-curve fit of R^2^ ≈ 0.99 in approximately 32,000 ART cycles (51), and has been implemented in routine clinical practice for over two years in our center, supporting more than 100,000 real-world assessments. It has also been promoted in multiple hospitals and health-examination institutions across China, with consistently positive clinician and patient feedback.

Ongoing work focuses on continual model updating with new real-world data, expansion to longitudinal AMH modeling to estimate decline velocity, and adaptation for broader female populations, including women with comorbidities, as well as integration with systemic health metrics such as cardiovascular markers, bone density, and metabolic aging to build a more comprehensive female health index.

The clinical applications are extensive. For fertility counseling, the reserve score informs optimal timing for conception or oocyte cryopreservation, whereas for women receiving chemotherapy or other gonadotoxic treatments, it supports ovarian risk stratification (52). In midlife women, the forecasted age of perimenopause can guide anticipatory care for symptom management and comorbidity prevention (27). Importantly, beyond prediction alone, this framework enables the establishment of ovarian endocrine age–based reference ranges for key biomarkers. By replacing purely chronological or population-based intervals with physiology-aligned benchmarks, it becomes possible to address the long-standing problem of excessively wide “normal” ranges caused by substantial inter-individual variability in ovarian reserve, thereby strengthening precision endocrinology, precision nutrition, and individualized preventive care.

**Figure 3:**
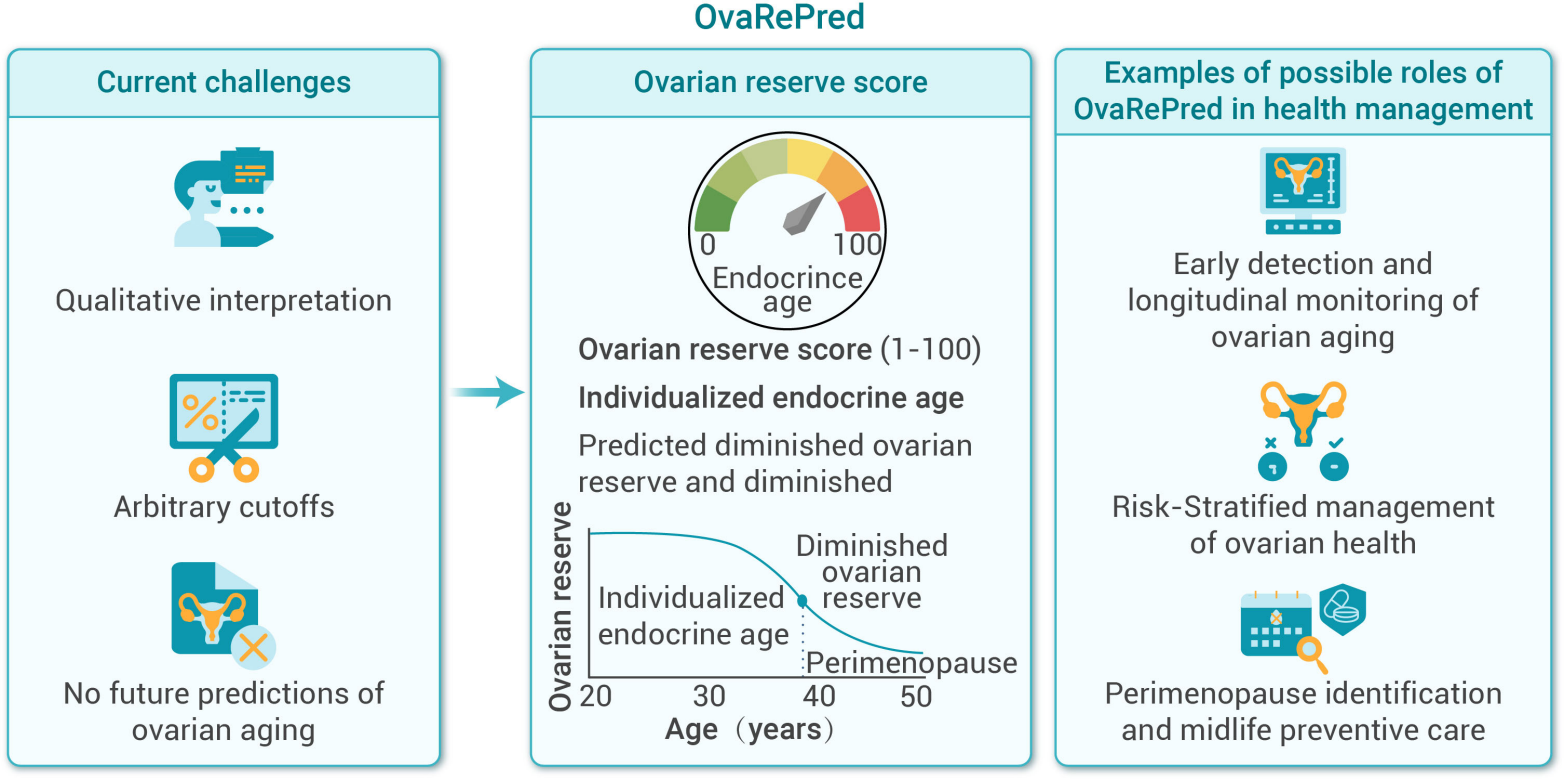
OvaRePred (HerTempo): personalized ovarian reserve evaluation and its role in health management. The schematic first highlights key challenges in ovarian reserve assessment, such as qualitative interpretation, reliance on arbitrary cutoffs, and the inability to predict future ovarian aging. It then presents OvaRePred (HerTempo), which generates an individualized ovarian reserve score (1–100) and endocrine age, allowing accurate prediction of diminished ovarian reserve and perimenopause onset. Clinical applications of OvaRePred (HerTempo) include early detection and longitudinal monitoring of ovarian aging, risk-stratified management of ovarian health, and timely identification of perimenopause to support targeted midlife preventive interventions.

In terms of usability and real-world impact, embedding OvaRePred (HerTempo) into consumer-facing digital platforms such as health apps and wearable systems would empower women to monitor reproductive health more regularly and make informed life and career decisions, while integration with electronic medical records and collaboration with health-tech companies could further facilitate scalable clinical implementation. Collectively, OvaRePred (HerTempo) represents a transition from reactive fertility management toward proactive, data-informed reproductive planning (50), and related research on endocrine age–based reference frameworks is ongoing, with supporting data to be reported in forthcoming publications. **Figure 3** schematically illustrates several key aspects of OvaRePred (HerTempo)’s personalized ovarian reserve evaluation and its role in health management, representing only a portion of its broader capabilities.

### 3.2 PCOSt: three-item PCOS triage at population scale

PCOS is among the most common endocrine and metabolic disorders affecting women of reproductive age, with a global prevalence ranging from 6% to 20% (53–58). Beyond reproductive issues, PCOS is increasingly recognized as an important early stage of various metabolic, cardiovascular, and psychological conditions, including type 2 diabetes, cardiovascular disease, hypertension, obesity, sleep apnea, depression, and anxiety (55, 59–61). These wide-ranging systemic consequences underscore the urgent need for scalable, efficient screening strategies capable of identifying high-risk women earlier and at a population level.

PCOSt (demo: http://121.43.113.123:8888/) is a streamlined, AI-driven screening tool created using extensive reproductive-center datasets to rapidly identify women at high risk for PCOS. Unlike the conventional Rotterdam criteria, which require ruling out other endocrine disorders (e.g., thyroid dysfunction, hyperprolactinemia, congenital adrenal hyperplasia) followed by confirmation of at least two of three criteria—oligo- or anovulation, hyperandrogenism, and polycystic ovarian morphology via ultrasound (46)—the PCOSt model simplifies screening significantly. The traditional Rotterdam criteria necessitate multiple assessments and repeated clinical visits, making them unsuitable for large-scale screening initiatives.

The initial development of PCOSt incorporated four predictors—AMH, upper limit of menstrual cycle length (UML), body mass index (BMI), and androstenedione (A_4_)—to capture biochemical hyperandrogenism alongside ovarian morphology and ovulatory dysfunction (62). During subsequent model refinement and external validation, we systematically evaluated the incremental contribution of A_4_ and found that its removal did not result in statistically significant differences in discrimination (AUROC) or calibration performance (63). This finding is biologically plausible, as AMH and A_4_ are moderately correlated in PCOS populations, reflecting overlapping pathophysiological signals of follicular excess and androgen activity (64). Guided by the principle of model parsimony and the goal of maximizing scalability in primary care and telemedicine settings, we therefore adopted the simplified three-variable model (AMH, UML, BMI) as the primary screening framework, while retaining the four-variable version as an extended option where androgen assays are routinely available. Built from 11,720 clinically diagnosed cases, the PCOS-3 model demonstrates excellent performance, with AMH emerging as the dominant contributor (47.0% main effect; 58.9% total effect), followed by UML (34.5% main effect; 46.2% total effect) and BMI (4.3% main effect; 7.9% total effect). In external validation, the model achieved an AUROC of 0.841 (95% CI 0.826–0.856) with strong calibration and probability–severity alignment (63). All predictions were benchmarked directly against Rotterdam-defined diagnoses assigned by reproductive endocrinologists, ensuring clinical relevance and real-world applicability, and risk thresholds can be flexibly adapted for challenging contexts such as adolescent screening.

Operationally, PCOSt is not intended to replace specialist diagnosis but to function as a scalable triage and risk-identification tool. It is particularly suitable for primary care, telemedicine, school-health or occupational screening programs, and digital health ecosystems where full Rotterdam assessment may not be immediately available. Emerging minimally invasive AMH measurement technologies, including finger-prick sampling or menstrual-blood-based testing, further enhance feasibility for population deployment (24, 25). Importantly, predictive thresholds within PCOSt can be adapted to address historically challenging diagnostic scenarios—such as adolescents, early phenotypic PCOS, or under-recognized metabolic-risk PCOS subtypes—thereby supporting earlier intervention and clinical referral.

**Figure 4:**
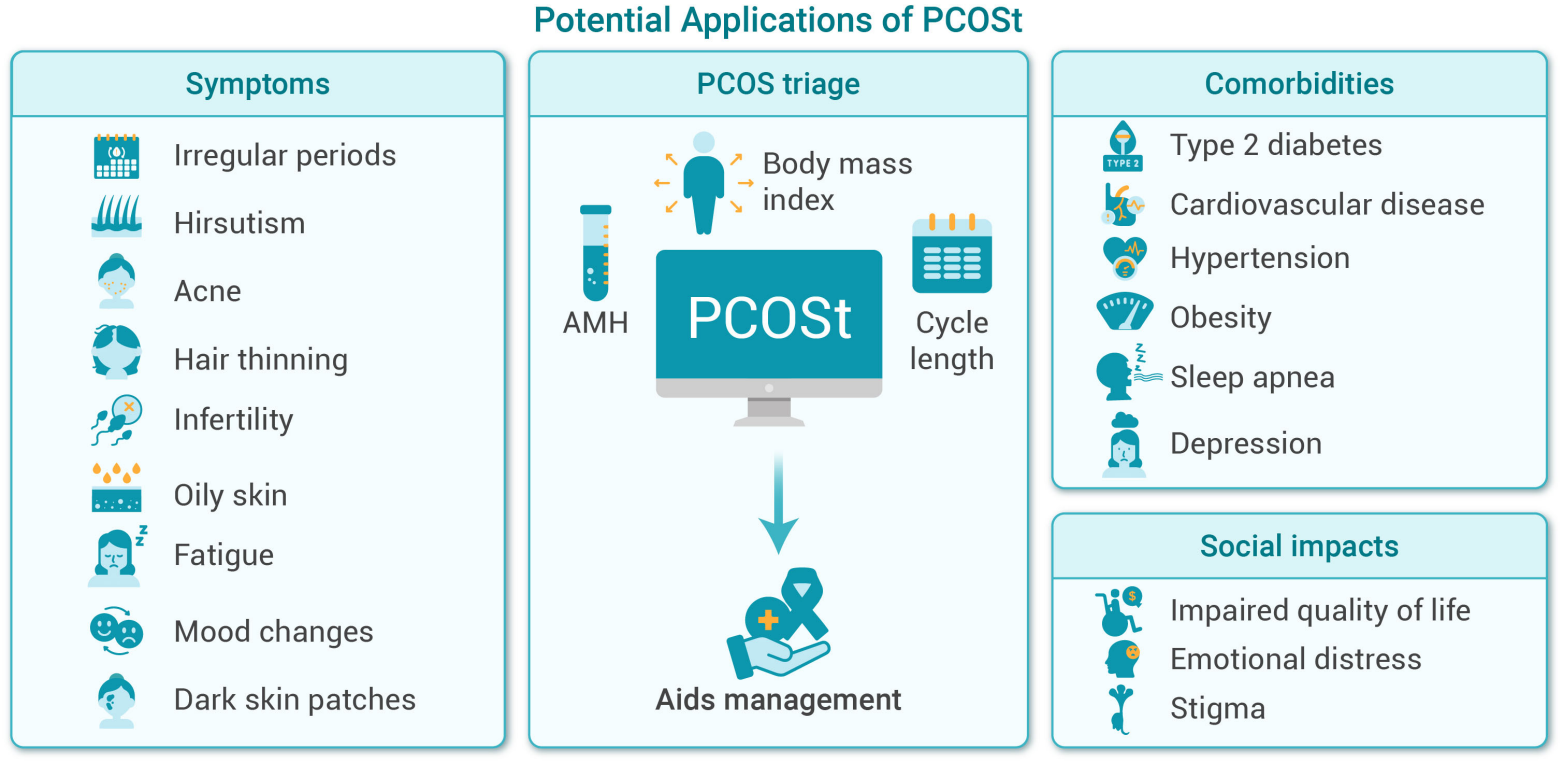
PCOSt: integrative approach to PCOS diagnosis and management. This figure outlines the potential applications of the PCOSt tool in clinical management. It integrates key symptoms, biochemical and clinical indicators for PCOS triage, and addresses associated comorbidities and social impacts, ultimately supporting improved diagnosis and comprehensive patient care.

Looking forward, integration of PCOSt with advanced AI technologies—such as ultrasound-based phenotyping using convolutional neural networks, multimodal risk classifiers incorporating metabolic and lifestyle factors, and phenotype-specific predictive models—may further enhance diagnostic granularity. Embedding PCOSt within national screening frameworks or adolescent health initiatives has the potential to substantially improve early diagnosis, personalized management, and long-term health outcomes worldwide. The integrative role of PCOSt in PCOS identification, stratification, and care pathways is illustrated in **Figure 4**.

### 3.3 POvaStim: algorithmic FSH dosing for controlled ovarian stimulation

In ART, selecting the optimal dose of FSH is essential for retrieving an adequate number of oocytes while ensuring their developmental potential (65). However, ovarian response varies widely among individuals due to differences in age, ovarian reserve, and hormonal profiles (66). Currently, FSH dosing largely depends on physician experience, which can lead to inconsistent and suboptimal outcomes. There is a clear need for AI-driven tools that enable data-informed, individualized dosing decisions.

POvaStim (demo: http://fsh.vecverse.com) is an AI-based clinical decision support tool designed to optimize gonadotropin dosing during COS in ART (38, 40). Accurate FSH dosing is critical: underdosing may cause poor follicular response or cycle cancellation, while overdosing increases the risk of ovarian hyperstimulation syndrome (OHSS), raises treatment costs, and heightens patient burden (67). POvaStim employs a two-step framework:

Step 1: Predict number of oocytes retrieved.

Baseline Model 1 (Day 2 predictors) is driven predominantly by AMH (main effect 90.2%), with smaller contributions from AFC (3.6%), FSH (1.2%), and age (0.3%). Day 6 Model 2 further adjusts prediction by incorporating Δ-inhibin B using Day 2 and Day 6 markers.

Step 2: Model ovarian sensitivity and recommend FSH dosing.

Model 3 predicts starting FSH dose using day 2 parameters. AMH is the principal determinant (main effect 83.5%; total effect 86.0%), followed by AFC (main 7.5%; total 9.9%), with smaller contributions from basal FSH (main 1.5%; total 2.4%) and age (main 1.5%; total 2.5%).

Model 4 predicts dose adjustment on menstrual cycle day 6, where Δ-inhibin B becomes the dominant contributor (main 48.9%; total 50.8%), followed by AMH (main 30.0%; total 31.8%), AFC (main 12.7%; total 14.5%), and age (main 2.0%; total 3.2%).

Together, both model 3 and model 4 models explain >90% of variability in ovarian sensitivity, demonstrating high predictive power and clinical interpretability (40). In a case–control study, junior physicians using POvaStim achieved outcomes comparable to senior physicians, suggesting its potential to bridge experience gaps and standardize decision-making.

**Figure 5:**
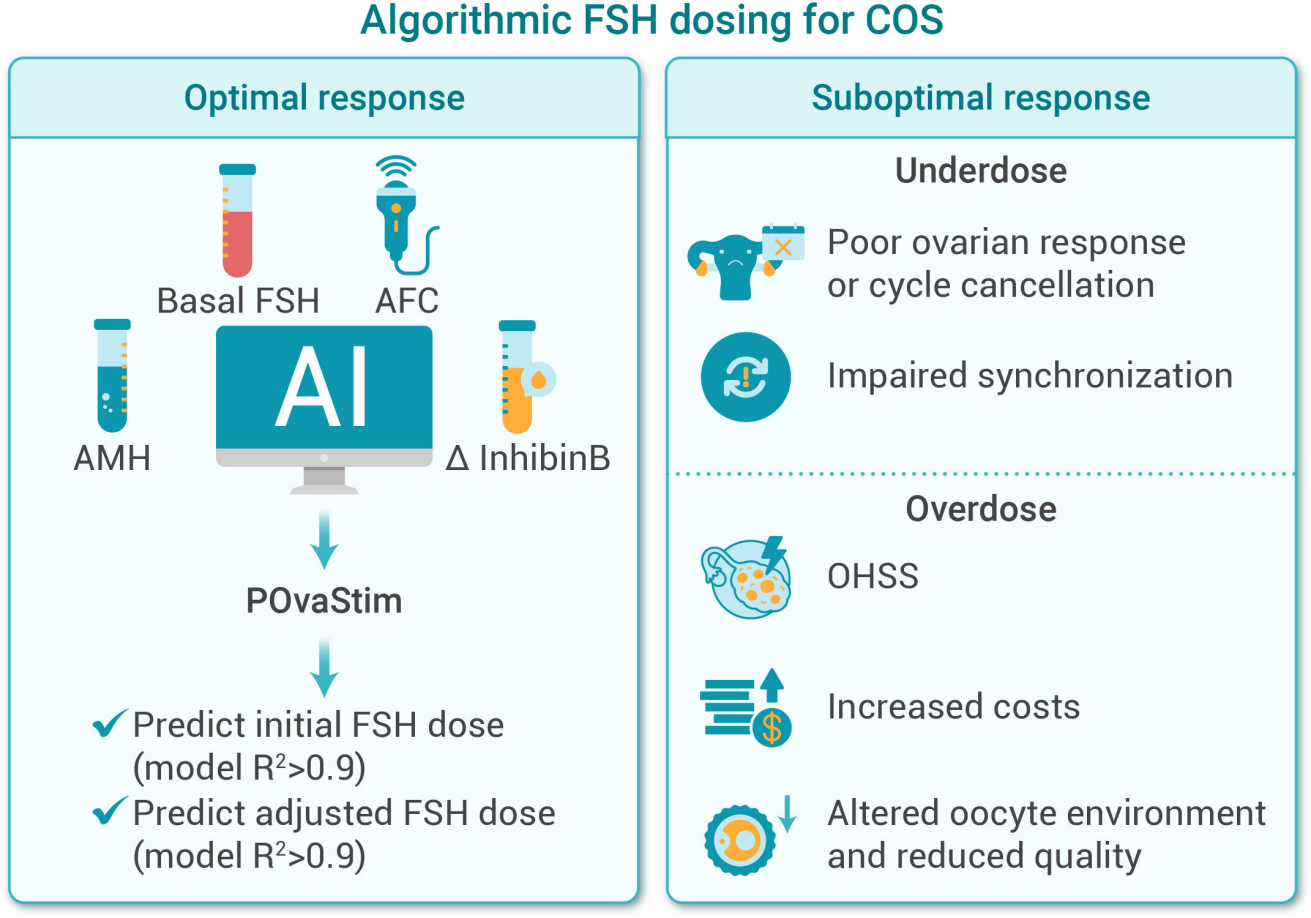
POvaStim: AI-based FSH dosing optimization in COS. This figure illustrates the AI-driven POvaStim tool for algorithmic FSH dosing in controlled ovarian stimulation (COS). Using basal hormone levels and AFC (antral follicle counts), POvaStim predicts initial and adjusted FSH doses to achieve optimal response, while addressing risks of underdose and overdose, including poor response, OHSS, and reduced oocyte quality.

Beyond supporting less experienced clinicians, POvaStim offers seasoned physicians a data-driven reference point, complementing their clinical judgment. However, as inhibin B is not yet routinely monitored, the dose adjustment model requires further real-world validation. Ultimately, POvaStim aims to enhance—not replace—clinical decision-making by translating complex biological data into actionable insights. With broader validation, its ovarian sensitivity–based algorithm could also extend to other COS protocols and fertility preservation strategies. **Figure 5** presents a schematic overview of AI-guided algorithmic FSH dosing for optimized controlled ovarian stimulation using POvaStim.

## 4 Future outlook: building an “ovarian intelligence” ecosystem

### 4.1 Ovarian intelligence: extending AI applications beyond ART

As AMH and inhibin B are increasingly integrated into artificial intelligence (AI) models, reproductive medicine is entering a new era of Ovarian Intelligence. Tools such as OvaRePred (HerTempo), PCOSt, and POvaStim, originally developed by reproductive medicine team to enhance the precision and efficiency of ART, have shown broader potential well beyond IVF. These AI systems are poised to become core components of proactive, personalized, and continuous women’s health management across the lifespan.

OvaRePred (HerTempo) enables accurate prediction of ovarian reserve and perimenopause timing, empowering informed decisions about fertility planning and menopausal health (47, 50, 68, 69). PCOSt offers early, data-driven screening and phenotyping of PCOS, helping identify metabolic and cardiovascular risks before clinical manifestation (50, 70). POvaStim leverages a model of ovarian sensitivity to recommend individualized FSH starting doses and mid-cycle adjustments, improving safety and effectiveness in ovarian stimulation (50, 70, 71). Together, these tools form an integrated AI chain—from risk screening to therapeutic guidance—offering comprehensive support for both reproductive and systemic health. More importantly, they are well-positioned for integration into broader health and wellness platforms.

### 4.2 Complementary modalities: integrating urinary and serum biomarkers

Emerging urinary hormone technologies measuring estrone-3-glucuronide (E3G) and pregnanediol-3-glucuronide (PDG) enable convenient, non-invasive, daily tracking of estrogen dynamics, ovulation timing, and luteal adequacy, and are particularly valuable for natural conception optimization (72–74). By contrast, serum-based AMH and inhibin B provide more stable information that supports ovarian reserve assessment, endocrine aging forecasting, clinical risk stratification, and interventional guidance. Rather than competing approaches, these modalities are inherently complementary. Integrating E3G/PDG data into AMH- and inhibin B-anchored AI frameworks offers a multimodal pathway in which long-term “capacity” signals from serum biomarkers are dynamically enriched by high-resolution, real-time functional information from urinary hormones. This convergence would extend ovarian intelligence tools from static prediction to continuous, at-home monitoring, enabling more precise fertility management, earlier preventive intervention, and fully personalized, lifecycle-oriented reproductive care.

### 4.3 From AI-assisted analytics to fully adaptive intelligence

It is important to differentiate what AI-enabled ovarian intelligence currently delivers from where the field is evolving. At present, most clinically deployed systems are built on rigorously validated statistical models derived from large-scale real-world datasets. These are predominantly “small models”—transparent, interpretable, computationally lightweight, and already proven in clinical environments. Their low resource demand and clear mechanistic logic make them highly suitable for routine deployment and also easy to integrate as trusted analytical modules within broader AI ecosystems or large foundation models. Looking ahead, however, the trajectory is moving toward increasingly advanced artificial intelligence, including multimodal learning that integrates hormonal, imaging, behavioral, genetic, and immunologic data (75, 76); longitudinal modeling to characterize endocrine aging dynamics; deep-learning–based ovarian morphology interpretation; and autonomous therapeutic optimization engines. Thus, while current platforms operate at the pragmatic interface between statistics and AI, they establish a robust, explainable, and scalable foundation for progressive evolution toward fully adaptive, continuously learning healthcare systems in reproductive medicine.

### 4.4 Digital health integration and the rise of nutritional AI

In the near future, this ecosystem will increasingly converge with digital health tools, behavioral data streams, and personalized nutritional strategies. By linking OvaRePred (HerTempo) with cycle-tracking platforms, wearable devices, home-based sampling, and lifestyle logs, continuous ovarian monitoring becomes feasible, enabling early identification of accelerated decline trajectories. Critically, the development of ovarian endocrine age–based reference ranges provides a physiological benchmark for interpreting hormonal and metabolic biomarkers, helping overcome the challenge of excessively wide “normal” ranges driven by individual variability. Anchoring nutritional decision-making to endocrine age, rather than chronological age alone, creates a foundation for precision nutrition, allowing targeted supplementation at the right timing and magnitude.

In this integrated framework, PCOSt can support subtype-specific nutritional guidance—for example, recommending tailored interventions such as inositol, probiotics, vitamin D, or metabolic-supportive nutrients for distinct PCOS phenotypes to improve endocrine and metabolic stability (77, 78). Likewise, ovarian sensitivity insights from POvaStim may extend beyond treatment populations to generally healthy women, informing individualized responses to functional supplements such as CoQ10, DHEA, or antioxidant regimens. Collectively, this signals the arrival of “Nutritional AI,”where supplementation strategies are no longer generic but dynamically personalized based on AI-derived biomarker intelligence. For the nutrition and health industry, integration with Ovarian Intelligence platforms offers an entirely new paradigm for precision product development, efficacy monitoring, and meaningful consumer engagement.

### 4.5 Technical foundations: standardization, POCT, and platform harmonization

From a technical standpoint, platform harmonization remains key to scalability. Cross-assay AMH conversion tools are emerging, but full standardization will require assay vendors to share batch-level calibration metadata (79). A collaborative industry framework is essential to ensure the stability and interoperability of AI models. On the diagnostic front, point-of-care testing (POCT) for AMH and inhibin B is progressing rapidly, with microfluidic chips and menstrual blood-based sampling poised to bring hormone tracking into the home (24, 25).

### 4.6 Longitudinal modeling, precision phenotyping, and oncology applications

Even more exciting is the integration of longitudinal biobanks and behavioral datasets, enabling dynamic AI modeling beyond static diagnostics. With repeated hormone measurements over time, these models will reveal trajectory-based insights—such as the rate of AMH decline—allowing early identification of accelerated reproductive aging. As models evolve, they will also incorporate genomic and immune layers (e.g., AMHR2/FSHR polymorphisms, anti-AMH antibodies, inflammatory markers), enhancing precision in complex conditions like autoimmune POI, endocrine-resistant infertility, and PCOS variants. Even in female cancer patients, anti-tumor treatments directly destroy follicles and disrupt the ovarian microenvironment by inducing DNA damage, oxidative stress, and vascular damage in follicles, ultimately leading to diminished fertility and endocrine dysfunction. The extent of this damage is closely correlated with the patient’s age, treatment type, and dosage. OvaRePred (HerTempo) demonstrates significant value in this context: Firstly, it enables baseline assessment of ovarian reserve before treatment, assisting physicians and patients in formulating personalized fertility preservation strategies. Secondly, post-treatment, through regular monitoring of ovarian reserve changes, OvaRePred (HerTempo) provides a basis for early intervention, allowing timely detection and management of treatment-induced early ovarian insufficiency, thereby maximizing the protection of the patient’s fertility potential.

Furthermore, the predictive capabilities of OvaRePred (HerTempo) extend to the comprehensive health management of cancer patients. By predicting future perimenopause onset timing, physicians can proactively intervene to assess and manage systemic risks associated with declining ovarian function, such as osteoporosis, cardiovascular disease, and metabolic syndrome. This facilitates the development of more holistic and personalized health management plans for patients. In summary, OvaRePred (HerTempo) not only holds broad promise in assisted reproduction and fertility planning, but its potential in fertility assessment and overall health management for female cancer patients also warrants further exploration and promotion.

### 4.7 Heterogeneity considerations: clinical phenotype, ethnicity, and assay platforms

Biological and technical heterogeneity must be carefully addressed for the clinical translation of ovarian intelligence tools. Clinical phenotype is a major source of variability: women with PCOS present markedly elevated AMH and distinct inhibin B dynamics compared with normo-ovulatory populations, potentially affecting probability estimates, calibration, and thresholds. Ethnicity and population background may also influence ovarian aging trajectories and biomarker distributions, underscoring the need for large, multi-center, multi-ethnic validation to ensure generalizability. In addition, assay platform variability— including calibration differences, inter-assay drift, and incomplete standardization—remains a key technical challenge. Although cross-platform conversion tools and improved automation are helping mitigate these issues, robust harmonization strategies, transparent assay reporting, and stratified modeling will be essential to ensure reliable, equitable, and scalable deployment of AI systems based on AMH and inhibin B.

### 4.8 Toward a full-spectrum ovarian intelligence ecosystem

Ovarian Intelligence is not just a clinical assistant; it is a navigational system for every woman’s reproductive journey. From fertility preservation planning to individualized perimenopause management; from adolescent risk screening to post-oncologic ovarian recovery monitoring; from PCOS-targeted metabolic interventions to personalized menopausal support—AMH and inhibin B–driven AI tools are continuously expanding their clinical and preventive value.

**Figure 6:**
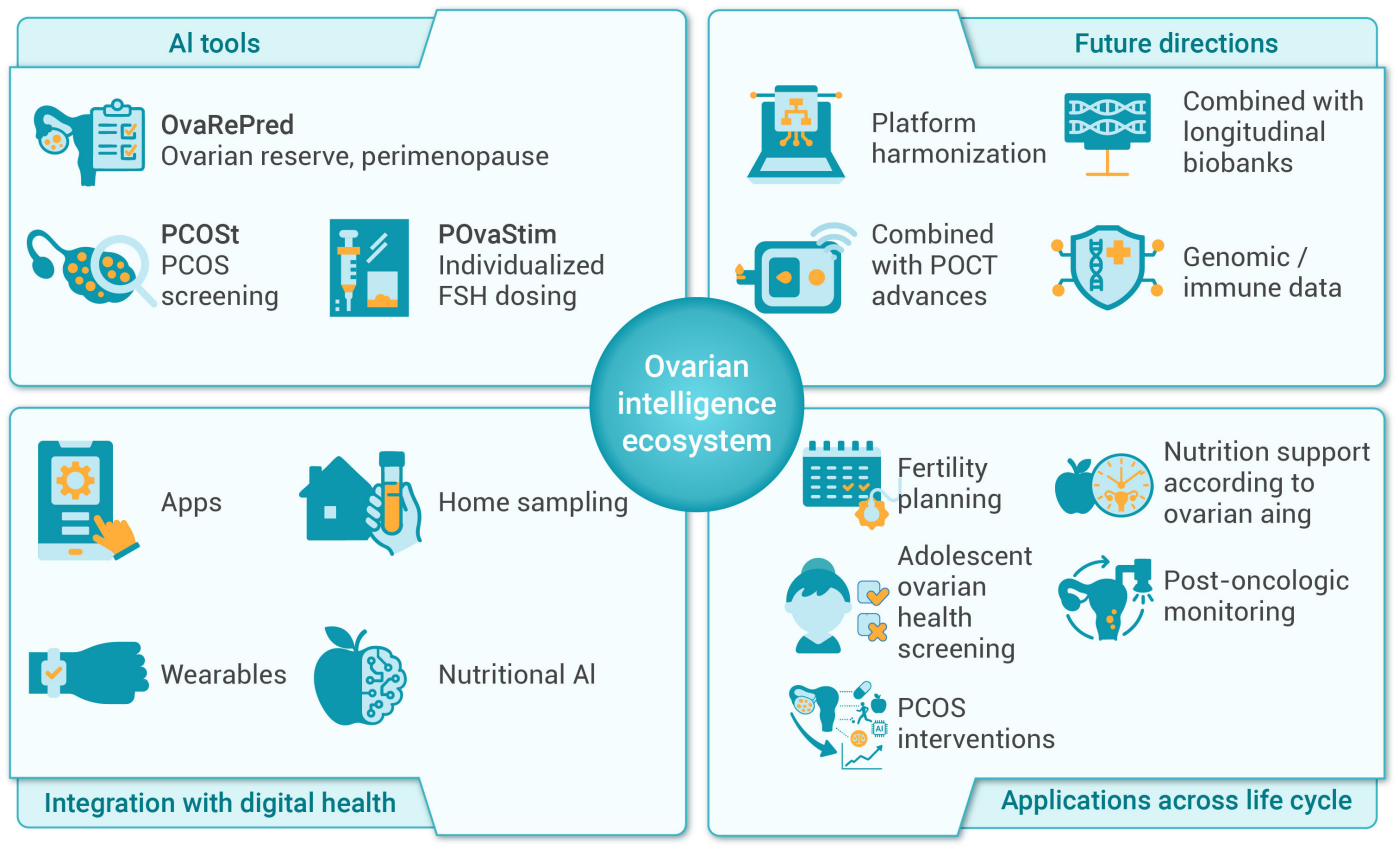
Comprehensive ovarian intelligence ecosystem and future directions. This diagram depicts the ovarian intelligence ecosystem, highlighting key AI tools—OvaRePred (HerTempo), PCOSt, and POvaStim—and their integration with digital health technologies such as apps, wearables, home sampling, and nutritional AI. Future directions include platform harmonization, biobank integration, genomic data incorporation, and more. The ecosystem supports various applications across the reproductive life cycle, including fertility planning, adolescent screening, PCOS intervention, nutrition, post-oncologic monitoring, among others. The illustrated applications represent a subset of the broader potential uses.

Ultimately, these tools will give rise to a full-spectrum, multi-scenario, population-wide AI ecosystem that bridges diagnosis, prediction, intervention, follow-up, and functional nutrition. They will redefine women’s healthcare by shifting the paradigm from reactive care to proactive, personalized, and intelligent health optimization. The Ovarian Intelligence Ecosystem, encompassing AI tools, digital integration, and lifecycle applications, is depicted in **Figure 6**.

## 5 Conclusion

Artificial intelligence is transforming reproductive medicine by translating hormone signals like AMH and inhibin B into personalized insights. Tools such as OvaRePred (HerTempo), PCOSt, and POvaStim enhance fertility care through prediction, screening, and individualized treatment. Looking ahead, these models hold great promise for integration with nutrition, digital health platforms, and point-of-care diagnostics to enable proactive, lifespan-based health management. As they evolve to incorporate longitudinal, genetic, and immunologic data, such AI systems may empower both clinicians and women with timely, tailored guidance—shifting reproductive health from reactive care to intelligent, preventive optimization.
